# Extensive screening of microRNA populations identifies hsa-miR-375 and hsa-miR-133a-3p as selective markers for human rectal and colon cancer

**DOI:** 10.18632/oncotarget.25535

**Published:** 2018-06-05

**Authors:** David Weber, Laurence Amar, Daniel Gödde, Christian Prinz

**Affiliations:** ^1^ Lehrstuhl für Innere Medizin 1, University of Witten, 42283 Wuppertal, Germany; ^2^ Institut des Neurosciences Paris-Saclay (Neuro-PSI)-CNRS UMR 9197, Université Paris-Saclay/Paris-Sud, 91405 Orsay, France; ^3^ Institut für Pathologie und Molekularpathologie, Helios Universitätsklinikum Wuppertal, 42283 Wuppertal, Germany

**Keywords:** differential expression, colorectal cancers, Illumina sequencing, matched tumor and normal tissues, hsa-miR-375 and hsa-miR-133a-3p

## Abstract

MicroRNAs (miRNAs) are ∼22-nt molecules exerting control of protein expression in cancer tissues. The current study determined the full spectrum of miRNA dysregulation in freshly isolated human colon or rectal cancer biopsies as well as in controls of healthy adjacent tissue (total of *n* = 100) using an Illumina sequencing technology. In this work, we aimed to identify miRNAs that may serve as future marker to discern between these two subtypes. DESeq2 analysis revealed 53 significantly dysregulated miRNAs in colon cancer, 67 miRNAs in rectal cancer, and 97 miRNAs in both at a *P*_adj_ value < 0.05 and ≥ 10 read counts. 65% of miRNAs were upregulated in colon as well as rectal cancer. Highest significant dysregulation (*P*_adj_ < 0.00001) was detected for hsa-miR-21-5p, -215-5p and -378a in both colon and rectal cancer. Among the group of miRNAs with *P*_adj_ < 0.05 and more than 2-fold expression differences, hsa-miR-375 was detected in rectal cancer only, and hsa-miR-133a-3p only in colon cancer. Receiver operating characteristic (ROC) analysis confirmed highly distinct sensitivities for hsa-miR-375 to detect rectal cancer (area under the curve (AUC): 0.9), while hsa-miR-133a-3p (AUC: 0.89) had the highest sensitivity for detecting colon cancer. We conclude that hsa-miR-375 and hsa-miR-133a-3p may serve as new markers of rectal or colon cancer and should be further investigated to search for different etiologies of colorectal cancer.

## INTRODUCTION

Colorectal cancer (CRC) is globally a leading cause of cancer-related death [1]. Surgery, i. e., hemicolectomy in colon cancer or total mesorectal excision with extended lymphadenectomy, is the only curative option in locally confined stages of cancers and is reasonable when the tumor has exceeded the mucosal layer [2]. Tumors that are limited to the intestinal wall without lymph node invasion (Union for International Cancer Control (UICC) stage I–II) are primarily operated while higher stages are usually pretreated by radiation combined with a 5-fluorouracil-based chemotherapy (UICC stage III–IV) [3]. Since rectal cancer, in contrast to colon cancer, can be treated with radiotherapy without damaging adjacent organs, and also presents differential patterns of tumor growth and metastasis [3–5], biomarkers discriminating between the two cancer types would be of clinical importance. Also, stage diagnoses are usually established by looking for lymph node infiltration and distant metastasis via non-invasive techniques such as magnetic resonance imaging, computed tomography, or endoscopic ultrasound. However, these techniques do not always correctly predict the tumor stage, as evaluated by surgery later on, and thereby impair an adequate therapeutic decision-making. For example in endosonographic techniques, the specificity for the correct tumor stage ranges only at 80–86% [5]. Therefore, new molecular tools are of clinical interest for predicting the further course of disease, complications, or response to radiation and chemotherapy [6].

MicroRNAs (miRNAs) are non–protein coding ∼22-nucleotide ribonucleic acids (RNAs) that post-transcriptionally affect targeted protein-coding gene expression. In complex with Argonaute (AGO) protein, miRNAs mainly use seed sequences at the 5′ end to base pair with a target messenger RNA (mRNA) and induce mRNA translational repression and/or degradation (reviewed in [7]). The association of miRNAs with cancer development and progression suggests that a new and interesting predictive tool has emerged with the potential to serve as a marker [8–10]. MiRNA expression profiles can identify human gastrointestinal cancer better than mRNA expression profiles [11]. In 2005, Lu *et al.* presented a systematic expression analysis of 217 miRNAs from 334 samples of multiple human cancers and reported a general downregulation of miRNAs in some tumors compared with normal tissues [11]. Furthermore, they successfully classified poorly differentiated tumors using miRNA expression profiles. These findings, which were collected through a systematic analysis of human cancers, highlight the potential of miRNA profiling in cancer diagnosis.

Expression of miRNAs known to be differentially regulated in various cancer types has also been determined in colorectal cancer, and miRNA deregulation in primary colorectal tumors has been correlated with tumor stage and overall survival [12]. An interesting example is hsa-miR-21-5p. Higher levels of hsa-miR-21-5p in CRC tissues compared with normal colon tissue have been associated with poor prognosis and unfavorable therapeutic response, independently of well-established clinical predictors [13]. However, despite the fact that CRC is a leading cause of cancer-related death, only few studies have employed large screening assays to evaluate the expression profile of miRNAs in colon and rectal tumors [14, 15].

Recent studies have described the role of miRNAs as a screening biomarker especially in CRC. For example, when using a panel of six miRNAs obtained in tissue specimes of patients with CRC, the panel of hsa-miR-7, hsa-miR-93, hsa-miR-195, hsa-miR-141, hsa-miR-494, and hsa-let-7b, at a cut-off value of two deregulated miRNAs distinguished early relapsed CRC from non-early relapsed CRC, with a sensitivity of 76.6% and a specificity of 71.4%. When combining this panel with six clinicopathologic factors, that study showed the potential to improve the prediction of early relapse in CRC patients [16]. Also, in a Chinese population, a number of miRNAs seemed to be important in the diagnosis and prognosis of colorectal cancer using the method of small RNA deep sequencing. Upregulation of hsa-miR-18a-5p and hsa-miR-21-3p or downregulation of hsa-miR-133a-3p in adenoma and cancer tissues seemed to serve as an index for early screening of colorectal cancer [17].

MiRNAs seem to be promising diagnostic biomarkers for colorectal cancer; however, there were multiple studies with conflicting findings. Studies varied significantly in ethnicity of populations, use of endogenous controls, source of miRNAs (whole blood, serum and plasma) and methods of detection. In a literature review, Clancy *et al.* determined six circulating miRNAs, hsa-miR-18a-5p, hsa-miR-21-5p, hsa-miR-29a-5p, hsa-miR-92a-5p, hsa-miR-143-5p and hsa-miR-378-5p, which were most frequently found to be dysregulated in colorectal cancer. The authors state that further studies identifying the source of tumor derived miRNAs in circulation, including identification of exosomal miRNA content, were required [18].

In this study, we have evaluated full expression profiles of miRNAs in fresh tissues from colon and rectal cancers and corresponding normal mucosa of 50 patients. In particular we compared the expression of more than 300 miRNAs using Illumina next-generation sequencing. We identified miRNA expression profiles characteristic for colon and rectal cancers and confirmed miRNA expression differences obtained with this technique with a second unrelated technique, real-time quantitative polymerase chain reaction (RT-qPCR) for relevant miRNAs, thereby, revealing the potential of several miRNAs as new diagnostic biomarkers. In addition, we demonstrated that a small group among more than 1850 miRNA molecules, i. e. hsa-miR-378a, hsa-miR-21-5p, and hsa-miR-215-5p, are of special interest for CRC detection and may have predictive potential.

Due to the vast heterogeneity of expression of molecular markers among patients bearing the same cancer type, diagnosis and/or therapeutic response prediction should be easily available and should be based on profiling of only a few miRNAs. Thus, we aimed to identify a small group of selective miRNAs in colon vs. rectal cancer. Possible projections of the use of the selective miRNAs in the clinic include their use on biopsy samples from patients before and/or after treatment, not only as a differentiating biomarker for disease type (colon or rectal cancer), but especially to evaluate and predict the therapeutic response following successful radiation that is routinely performed in rectal cancer patients, as well as an improved clinical staging and prediction of metastasis development or overall survival.

## RESULTS

### Biometric analysis using DESeq2

**Figure 1:**
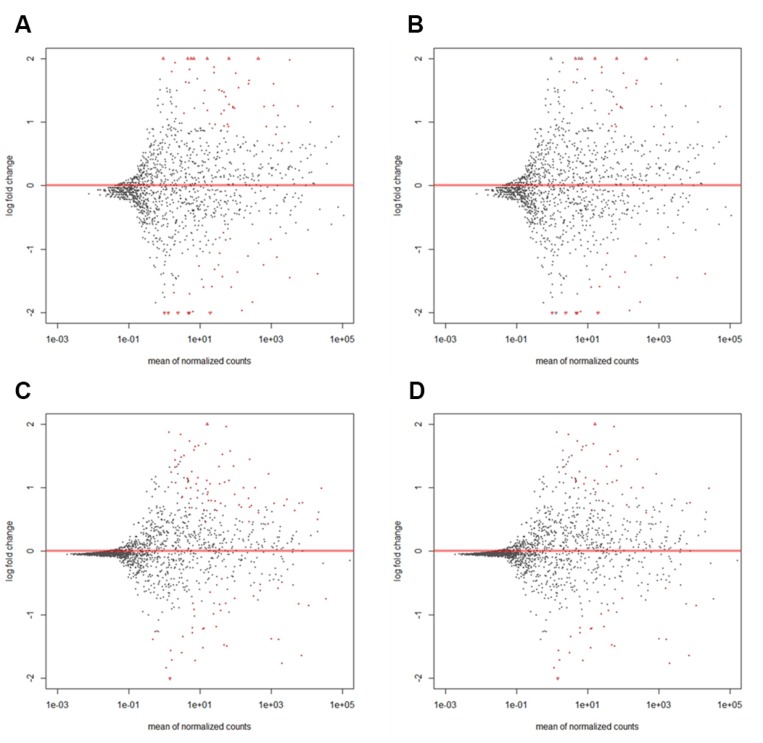
MA plots of differential miRNA expression in colon (**A**, **B**) or rectal cancer (**C**, **D**) at different statistical significance levels of *P*_adj_ < 0.05 (A, C) or *P*_adj_ < 0.01 (B, D). The figure shows the relative expression of single miRNAs (illustrated in single dots) of tumor vs. normal tissue (log2 fold change) plotted to normalized mean read counts. Dots are red for *P*_adj_ < 0.01 (B, D) or *P*_adj_ < 0.05 (A, C). Only miRNAs with more than 10 reads in average (10^1^) were considered in further investigation. Data points with a log2 fold change > 10e2 or < 10e−2 of tumor vs. normal tissue are plotted as open triangles pointing either up or down. A total of 15 colon and 35 rectal cancer tissues were investigated. Details are listed in Supplementary Table 1.

Biometrical analysis was performed using DESeq2 and results at different *P*_adj_ values (*P*_adj_ < 0.01 or *P*_adj_ < 0.05) are presented in Figure 1 and Supplementary Table 1. A total of 15 colon and 35 rectal cancer and control matched samples were investigated. Control matched samples were separate biopsies of the surrounding non-tumor tissue from identical patients. Mean miRNA expression differences (log2 fold changes) between tumor and normal tissues are shown on the y-axis, and mean miRNA expressions are shown on the x-axis. Out of a total of 1850 miRNAs identified in this assay, ∼320 miRNAs with more than 10 reads on average (10^1^) were considered for further investigation. This minimum threshold guarantees a reliable and accurate analysis. miRNAs expressed at lower mean read counts were not further investigated. Statistically significant differences in matched cancer vs. normal tissue are marked as red dots. At a *P*_adj_ < 0.01 and a mean expression threshold of 10^1^, a total of 34 miRNAs were significantly altered in colon cancer tissues: 21 were upregulated, and 13 were downregulated. In rectal cancer tissue at *P*_adj_ < 0.01, 37 miRNAs were significantly altered; 23 were upregulated and 14 downregulated. This finding indicates that ∼62% of expression differences concerned upregulation in the tumor tissue, in contrast to previous reports [11]. At a *P*_adj_ < 0.05 and a mean expression of 10^1^, statistical analysis using DESeq2 revealed a relatively small group of differentially expressed miRNAs for colon cancer (53 miRNAs), rectal cancer (67 miRNAs), or both types (97 miRNAs). Details are listed in Supplementary Table 1. Sequencing data was submitted to Sequence Read Archive (SRA) database, and will be available upon publication with accession number SRP132656 or with the following link: https://www.ncbi.nlm.nih.gov/sra/SRP132656 [19].

Details regarding the selective expression of these miRNAs are given in Supplementary Table 1. Among the group of miRNAs with statistically significant *P*_adj_ < 0.05 and more than 2-fold (log2 value of > 1 or < −1) expression differences, miR-375 was detected in rectal cancer only with a mean expression of 2002 read counts, a log2 fold change of -1.76 and a *P*_adj_ = 1.35E-09, while hsa-miR-133a-3p was detected in colon cancer only with mean expression of 620 read counts, a log2 fold change of -1.25 and a *P*_adj_ = 1.58E-02. Concerning the overall distribution (as presented in Supplementary Table 1), it appears that approximately 2/3 of the miRNA has a specific dysregulation for colon or rectal cancer, while only 1/3 of the miRNA has a similar expression pattern in both tumor types.

### Heatmap illustration of miRNA specific dysregulation in tumors

**Figure 2:**
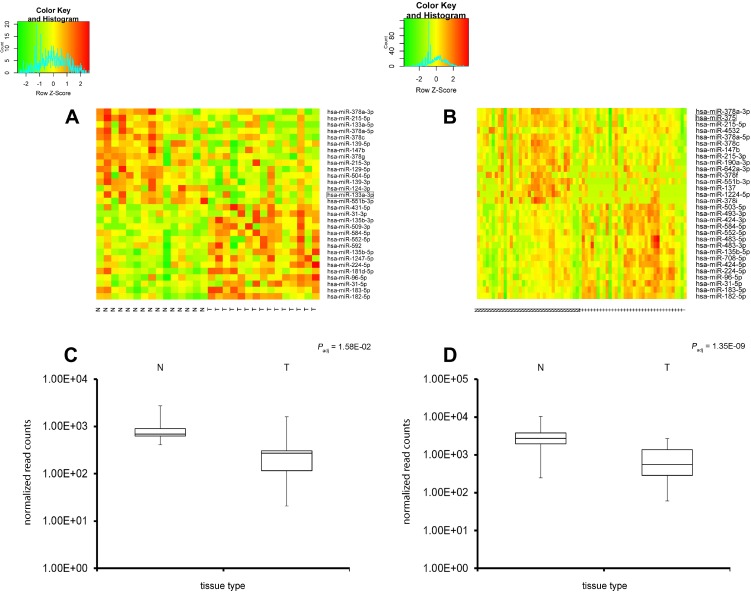
Expression screening of the miRNA molecules in colon and rectal adenocarcinoma (heatmap analysis) Heatmap of expression profiling of miRNAs in CRC; expression data for 30 highly differentially expressed miRNAs in colon (**A**, left side) or rectal cancer (**B**, right side). Red represents strong miRNA expression levels whereas green indicates miRNA downregulation; direct comparison of tumor (T) vs. normal tissue (N) can be visualized. An additional Box plot analysis is shown underneath the heatmaps of hsa-miR-133a-3p in colon cancer vs. normal tissue (**C**) and hsa-miR-375 in rectal cancer vs. normal tissue (**D**). The expression patterns show a clear distinction between normal and tumor tissues (colon or rectal cancer).

Expression of the thirty miRNAs identified by lowest *P*_adj_ values and higher fold-changes between matched tumor and normal tissues are visualized as heatmaps in Figure 2. Fifteen upregulated miRNAs and fifteen downregulated miRNAs are shown. Red represents strong expression levels in the specimens and green color indicates downregulation of miRNA. A direct comparison of tumor vs. normal tissue can be made by comparing the samples side by side. The specificity of hsa-miR-375 downregulation in rectal cancer is clearly visible (*P*_adj_ = 1.35E-09 in rectal cancer but *P*_adj_ = 3.82E-01 in colon cancer) similar to the specific downregulation hsa-miR-133a-3p in colon cancer (*P*_adj_ = 1.58E-02 in colon and *P*_adj_ = 9.05E-01 in rectal cancer). The heatmaps show that miRNAs could be used as markers to distinguish tumor from normal tissue. Based on miRNAs differentially expressed between normal tissue and rectal or colon cancer, respectively, cancer could be distinguished between each sample type with expression patterns differing clearly between benign tissues and colon or rectal cancer. Thus, miRNA expression classifies colorectal cancers and showed a clear difference from normal tissues.

### MiRNA patterns obtained by Illumina sequencing were confirmed by RT-PCR

**Figure 3:**
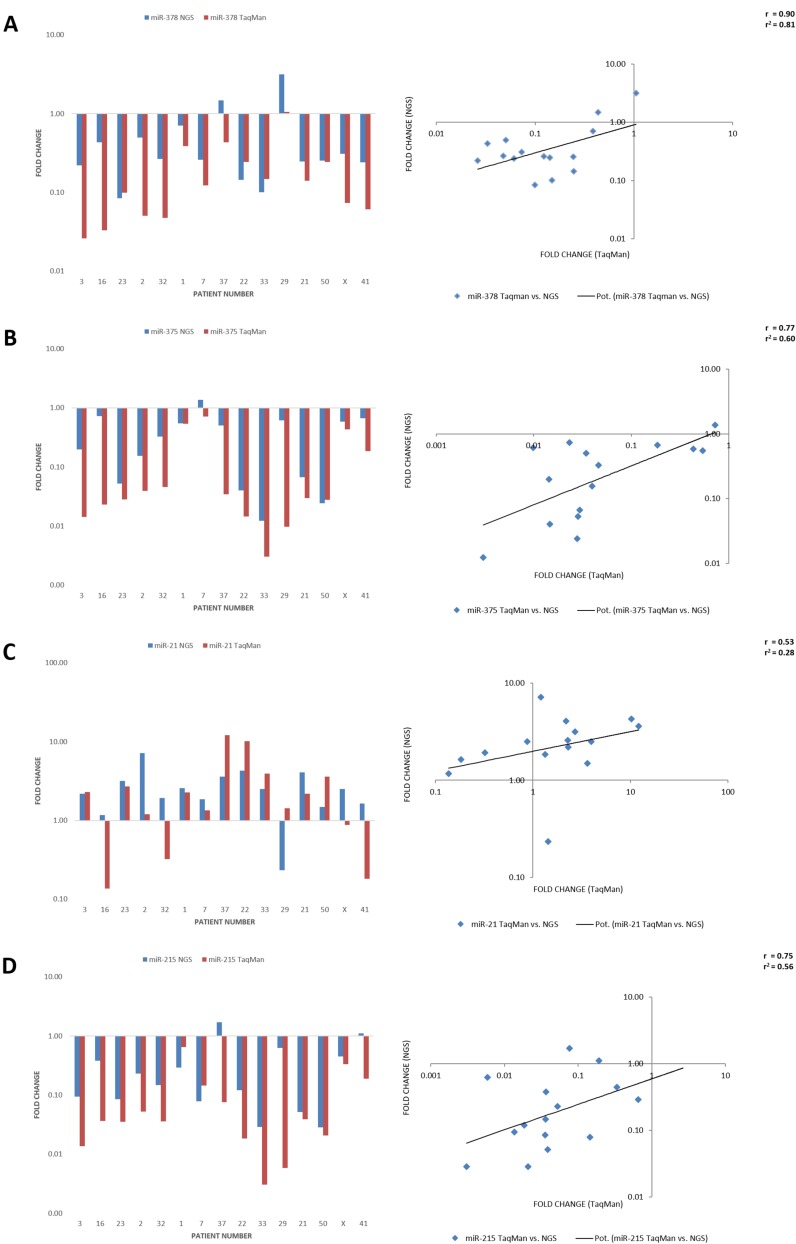
Comparison of miRNA expression levels obtained by NGS Illumina sequencing vs. TaqMan PCR. The results of the correlation are visualized as bar diagrams on the left and scatter diagrams with regression lines on the right comparing next generation sequencing (blue/y-axis) and TaqMan fold changes (red/x-axis) for hsa-miR-378a (**A**), hsa-miR-375 (**B**), hsa-miR-21-5p (**C**) and hsa-miR-215-5p (**D**). There was a good correlation of the NGS with the TaqMan results with a strong relationship for hsa-miR-378a (Pearson’s *r* = 0.9), hsa-miR-375 (*r* = 0.77) and hsa-miR-215-5p (*r* = 0.75), but only a moderate relationship for hsa-miR-21-5p (*r* = 0.53).

To validate the expression analysis by Illumina sequencing, we performed TaqMan analysis and determined the quantitative expression by PCR (Figure 3). Four miRNAs were selected based on lowest *P*_adj_ values and mean expression levels: hsa-miR-21-5p, hsa-miR-215-5p, hsa-miR-375 and -378a. A strong correlation was observed for hsa-miR-378a (Pearson’s *r* = 0.9), hsa-miR-375 (*r* = 0.77) and hsa-miR-215-5p (*r* = 0.75), underlining that Illumina sequencing is a useful and correct tool for this screen while TaqMan PCR can be regarded as a quick and inexpensive way to determine the expression of a single miRNA. In contrast, only a moderate relationship was observed for hsa-miR-21-5p (*r* = 0.53). In the case of hsa-miR-21-5p, a discrepancy was observed which may be explained by poor primer settings in the TaqMan PCR conditions. However, since hsa-miR-21-5p was not the primary focus of this work, the difference was not investigated in greater detail.

### Receiver operating characteristic analysis

**Figure 4:**
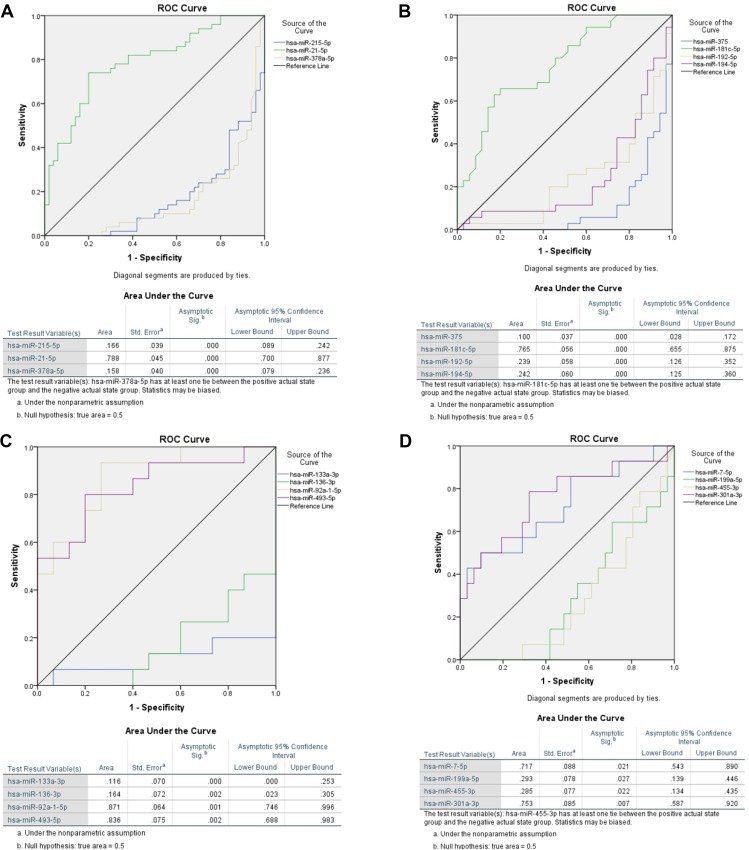
Receiver operating characteristic (ROC) analysis of miRNAs for all significant dysregulated miRNAs for colorectal cancer (**A**) as well as for rectal (**B**) and colon cancer (**C**) separately were performed and most promising candidates are shown here. Furthermore we calculated ROC curves for miRNAs with the highest potential to distinguish between colon and rectal cancer tissue samples (**D**). We selected three microRNAs miR-21-5p (AUC 0.79, 95% CI 0.70–0.88), miR-215-5p (AUC 0.83, 95% CI 0.76–0.91) and miR-378a (AUC 0.84, 95% CI 0.76–0.92) which were the most promising candidates for colorectal cancer. Each marker had an area under the curve (AUC) of ≥ 0.79 for detecting CRC selectively at a *P*_adj_ < 0.01 (A). ROC analysis also revealed that hsa-miR-375 had the best sensitivity for detecting rectal cancer, with an area under the curve (AUC) of 0.90 (95% CI 0.83–0.97) (B). Hsa-miR-133a-3p, in contrast, had the highest sensitivity for detecting colon cancer, with an AUC above 0.88 (95% CI 0.75–1.0) (C). Finally, when comparing colon cancer tissue directly to rectal cancer tissue, the ROC analysis indicated that hsa-miR-7-5p and hsa-miR-301a-5p had a fairly good specificity for detecting colon cancer (vs. rectal cancer) presence, but with an AUC only above 0.72 (D).

Receiver operating characteristic (ROC) analysis by using miRNA expression indicated that a combination of miRNAs allowed a distinction between colon and rectal cancer. Three miRNA molecules were highly sensitive and specific for detecting CRC: hsa-miR-378a, hsa-miR-215-5p, and hsa-miR-21-5p. Figure 4 illustrates that all markers had an area under the curve (AUC) of more than 0.79 for detecting CRC selectively. ROC analysis also revealed that hsa-miR-375 had the best sensitivity for detecting rectal cancer, with an area under the curve (AUC) of 0.90 (95% confidence interval: 0.83–0.97). Hsa-miR-133a-3p, in contrast, had the highest sensitivity for detecting colon cancer, with an AUC above 0.89 (95% CI confidence interval: 0.75–1.0). Finally, when comparing colon cancer tissue directly to rectal cancer tissue, the ROC analysis indicated that hsa-miR-7-5p and hsa-miR-301a-5p had a fairly good specificity for detecting colon cancer (vs. rectal cancer) presence, but showed an AUC only above 0.72. Thus, rectal and colon cancers associate with specific miRNA deregulation, specifically of hsa-miR-21-5p, hsa-miR-215-5p, and hsa-miR-378a. Our data suggest that dysregulation of hsa-miR-375 and hsa-miR-133a is limited to rectal or colon cancer, respectively, and underline their potential to serve as a marker. Different miRNA expression patterns for rectal and colon cancers imply different etiologies.

## DISCUSSION

Based on the biometric analysis using DESeq2 at significant *p*-values of *P*_adj_ < 0.01, a total of 34 miRNAs were dysregulated in colon cancer, 21 upregulated and 13 downregulated. In rectal cancer, 23 were upregulated and 14 downregulated. These data are in accordance with previous observations indicating that about one third of miRNAs are downregulated in gastrointestinal cancers while two thirds are upregulated [20], but are in contrast to initial works presented by Lu *et al.* [11]. However, several miRNAs were expressed at very low expression levels, and could therefore not be regarded as reliable and reproducible markers. Thus, we aimed to determine the expression profile of miRNAs at a *P*_adj_ < 0.05 and a mean expression of ≥ 10^1^. Statistical analysis using DESeq2 revealed a relatively small group of differentially expressed miRNAs for colon cancer (53 miRNAs), rectal cancer (67 miRNAs), or both types (97 miRNAs). Details are listed in Supplementary Table 1. Strikingly, the DESeq2 calculation revealed a highly significant upregulation (*P*_adj_ < 0.00001) of three miRNA molecules in both colon as well as rectal cancer: hsa-miR-21-5p, hsa-miR-378a, and hsa-miR-215-5p. While hsa-miR-378a can be regarded as a relatively new biomarker, the other two molecules have been extensively characterized in this context.

Sun *et al.* (2016) [15] studied 44 pairs of samples of CRC and adjunct normal tissue using Solexa sequencing. In this work, a strategy aiming at lowering biases was applied by the ligation of 5’ adaptors to miRNA ends. This allowed them to identify 34 differentially expressed miRNAs. Amongst these miRNAs, hsa-miR-21-5p appeared as an abundant miRNA that was up-regulated in both types of CRC (Tumor/Normal ratio of 2.4), and hsa-miR-143, as an abundant miRNA that is down-regulated in both types of CRC (Tumor/Normal ratio of 0.6) [15], similar to our results. Shee *et al.* (2013) [14] characterized the miRNA transcriptome in a cohort including 88 CRC tumors with long-term follow-up by deep sequencing. They characterized low expression of hsa-miR-592 and high expression of hsa-miR-10b-5p and hsa-miR-615-3p were associated with tumors located in the right colon relative to the left colon and rectum and high expression of hsa-miR-615-3p [14].

In our study, hsa-miR-21-5p was highly expressed, similar to earlier investigations and showed statistically significant differences between tumor and normal tissue. Interestingly, elevated levels seem to have a certain prognostic significance besides the role as a biomarker. Previous studies have determined that hsa-miR-21-5p expression levels associate with disease-free and overall survival in CRC [13]. In our current work, hsa-miR-21-5p could not be determined as a predictor of overall survival, which may be explained by the smaller number of patients treated and the short observation period. Hsa-miR-21-5p functions in many cell types as an anti-apoptotic and pro-survival factor and plays a significant role in cancer biology [21–24]. Elevated circulating hsa-miR-21-5p levels have also been detected in hepatocellular carcinoma [25], osteosarcoma [26], and lung cancer [27]. In line with previous studies [28], our data support a key role for hsa-miR-21-5p in colorectal cancer, consistent with findings underlining its important role in cancer biology, even if it doesn’t seems to be specific for colorectal cancer but cancer in general.

Another study tested two cohorts of 197 colon cancer patients, using microarrays containing 389 miRNA probes [13]. The analysis revealed 37 miRNAs that were differentially expressed in stage II colonic adenocarcinoma compared with adjacent normal tissue. In one of the cohorts, hsa-miR-20a, hsa-miR-21-5p, hsa-miR-106a, hsa-miR-181b, and hsa-miR-203 were deregulated in tumor tissues, with high tumor-to-normal ratios, as well as being associated with poor overall survival. However, the prognostic relevance was confirmed in the validation set for only one of the candidates, hsa-miR-21-5p, and the clinical and biological implications of differential expression of the remaining miRNAs were unclear. Taking together our current results with those of previous groups, we note that hsa-miR-21-5p levels appear to be an important biomarker correlating with cancer stage, development of distant metastasis, and survival in colon as well as rectal cancer.

In the current study, expression of hsa-miR-378a was also found at highly significant *P* levels in both colon and rectal cancers. Previous reports using Western blot and quantitative RT-PCR analysis indicated that BRAF was downregulated by hsa-miR-378-5p in CRC cells [29]. Moreover, hsa-miR-378-5p was negatively associated with BRAF in CRC tissues compared to adjacent non-tumor tissues. These results demonstrate that downregulation of hsa-miR-378-5p promotes CRC cell growth by targeting BRAF and that restoration of their levels is a potentially promising therapeutic in CRC [29].

Our data further identified an important role for hsa-miR-215-5p in colon as well as rectal cancer. Hsa-miR-215 mediated targets/pathways based on translational immunoprecipitation expression analysis [30], and the CDX1–miR-215 axis seem to regulate CRC stem cell differentiation [31]. An evaluation and replication of miRNAs with disease stage and CRC-specific mortality have also been shown for hsa-miR-215 [32, 33]. Hsa-miR-215 is associated with inhibition of CRC relapse following radical surgery [34].

In contrast, Illumina expression analysis followed by ROC analysis revealed *selective* expression of hsa-miR-375 in rectal cancer and may be a new tool for understanding the differential cancer biology of tumor cells. Hsa-miR-375 was first identified as a pancreatic islet-specific miRNA regulating insulin secretion, and studies revealed that hsa-miR-375 is a multifunctional miRNA participating in pancreatic islet development, glucose homeostasis, mucosal immunity, lung surfactant secretion, and tumorigenesis [35]. Recently, hsa-miR-375 was found to be significantly downregulated in multiple types of cancer lines and to suppress core hallmarks of cancer by targeting several important oncogenes like AEG-1, IGF1R, and PDK1 [35]. Generally, a tumor-suppressive role for hsa-miR-375 in experimental cancer progression has been postulated; however, the mechanisms underlying the dysregulation of hsa-miR-375, its potential use in prognosis and diagnosis in clinical conditions, and the therapeutic prospects of hsa-miR-375 in cancer are still under investigation. Hsa-miR-375 also seems to affect colorectal cancer cell sensitivity to cetuximab by targeting PHLPP1 [36] and BRAF [29]. Strikingly, hsa-miR-375 was not detected in colon cancer at significantly dysregulated values. Overall, the highly significant dysregulation as well as the results from ROC analysis support a role as a biomarker of rectal cancer.

Recent studies suggest that miRNA hsa-miR-375 may have the potential to act as blood-based biomarker to monitor the activity and progression of disease in patients with ulcerative colitis. In a relatively small group patients were allocated to three disease groups (ulcerative colitis, *n* = 37; dysplasia, *n* = 2; colitis-associated cancer, *n* = 6). Analysis of variance was used to assess differences between the groups. Hsa-miR-375 was significantly upregulated in the colitis-associated cancer cohort (*p* = 0.0061) compared with active ulcerative colitis. Combining several miRNAs in a panel increased the capacity of the test to distinguish between colitis-associated cancer and different ulcerative colitis activity states [37]. Also, in an Egyptian cohort of 64 patients, the expression pattern of hsa-miR-375 and hsa-miR-760 were significantly downregulated in serum of colorectal cancer compared to controls, in line with our data [38].

Furthermore, in a very comprehensive British study, recent data indicate that the down-regulation of hsa-miR-375 in plasma and tissue is matched in CRC [39]. In that work, bioinformatics prediction revealed hsa-miR-375 association with some critical signal pathways in the development and progression of CRC. A total of 42 miRNAs were identified in cancer tissue, which were differentially expressed in patients and healthy individuals. The results indicated that for plasma sample, hsa-miR-375 and hsa-miR-206 were dysregulated and could discriminate CRC patients from healthy controls. For tissue samples, hsa-miR-375, hsa-miR-150, hsa-miR-125b and hsa-miR-126 were down-regulated. Thus, hsa-miR-375 was the only miRNA that was significantly down-regulated and correlated (with a correlation coefficient of *r* = 0.47) in both tissue and plasma samples [39]. Most strikingly, these findings are in line with our current results with even higher significance levels.

Reciprocally, Illumina expression analysis followed by ROC analysis point out an important role for hsa-miR-133a-3p for colon cancer. Previous reports have already detected a role for hsa-miR-133a in CRC: For example, a Turkish group reported that decreased expression of hsa-miR-133a correlated with poor prognosis in CRC patients [40, 41]. Hsa-miR-133a was shown to be significantly downregulated in primary colorectal cancer specimens compared with matched adjacent normal tissue [42]. Ectopic expression of hsa-miR-133a significantly suppressed colorectal cancer cell growth *in vitro* and *in vivo* and functions as a tumor suppressor. Cell-cycle analysis revealed that hsa-miR-133a induced a G0/G1-phase arrest, concomitant with the upregulation of the key G1-phase regulator p21, increased p53 protein and induced p21 transcription [42]. Thus, our findings are in accordance with previous results but suggest a specific role of hsa-miR-133a-3p for colon cancer (vs. rectal cancer).

Several studies have been published reporting the relationship between CRC and hsa-miR-133a and thus, a meta-analysis has already been performed in regard to the predictive value of hsa-miR-133a in digestive systems neoplasms. Five diverging studies were enrolled in this meta-analysis, mostly performed on analysis of formalin-fixed tissues. The pooled result showed that decreased expression of hsa-miR-133a predicted poor overall survival (OS) in solid cancer patients (HR = 1.73). These data are in accordance with our current results, however, it has to be emphasized that because of the limited overall amount of data used in this preliminary meta-analysis, additional studies are required to verify the poor prognosis of decreased hsa-miR-133a in solid tumors [43]. Our current work on freshly isolated tissue further underlines the importance of hsa-miR-133a in CRC, and suggests that decreased hsa-miR-133a expression may serve as a biomarker for colon cancer presence.

In summary, previous studies have evaluated an association of miRNA expression profiles with gastrointestinal cancers and interestingly, the miRNA expression profiles seemed to classify human gastrointestinal cancer better than did mRNA–protein expression profiles [11]. However, a full-spectrum screening assay of miRNA dysregulation in colon vs. rectal cancer has been lacking until the current investigation. Our data are largely in line with previous studies, although we investigated a smaller number but yielded hihly significant statistical values. Due to a good tissue quality in this study, we were able to determine the selective dysregulation of certain miRNAs in colon cancer (hsa-miR-133a-3p), rectal cancer (hsa-miR-375), or both (hsa-miR-21-5p, -215-5p and -378a). Overall, these molecules may be further investigated as biomarkers for CRC in blood, used to search for molecular miRNA targets and thereby for new therapies, and to understand the different etiology of rectal versus colon cancer.

## MATERIALS AND METHODS

### Study population and tissues

The investigated colorectal cancer tissues (*n* = 50) and corresponding adjacent normal tissue samples (*n* = 50) were freshly obtained from patients who underwent curative surgery, following a radiotherapy treatment in the case of rectal cancers, between 2012 and 2016 at the Helios Klinikum Wuppertal. Details about the patients are shown in Supplementary Materials (Supplementary Table 2). Fresh tissue samples (total of *n* = 100) were obtained by an experienced pathologist following informed patient consent. They were taken directly after surgery from the tumor and adjacent non-tumoral colorectal tissue which served as matched controls. Tissue samples were immediately frozen at –80° C. Total RNAs were extracted using QIAzol^®^ (Qiagen, Venlo, NL), according to the manufacturer’s instructions. Quality of extracted RNA was controlled by BioAnalyzer. RNA was accepted with a RNA integrity number (RIN) > 8.

### Construction of cDNA libraries

Briefly, libraries were built using Illumina TruSeq™ multiplexing method [44]. Small RNAs of 16–36 bp were separated from total RNA by denaturating (8M urea) polyacrylamide (15%) gel electrophoresis. An adenylated DNA oligonucleotide (3′ adapter) was ligated to the 3′ end of the RNAs and a chimerical DNA/RNA oligonucleotide (5′ adaptor) to the 5′ end of the RNAs. Ligation steps were followed by reverse transcription (RT) and polymerase chain reaction (PCR). After gel electrophoresis purification, cDNA libraries were run through a BioAnalyzer quality check (cDNA size and concentration) and were finally analysed on an Illumina NextSeq 500 sequencer at the Institute of Integrative Biology of the Cell Paris (I2BC).

### Data analysis

Data received from the sequencing platform were analysed and sequences were matched to their corresponding miRNAs using Galaxy (https://usegalaxy.org/) [45–47] and sRNAbench (miRBase 21, http://bioinfo5.ugr.es/srnatoolbox/srnabench/) [48]. The statistical analysis was performed with the statistical programming language R including DESeq2. DESeq2 is a R/Bioconductor package for differential gene expression analysis in count data from high-throughput sequencing assays based on the negative binomial distribution [49]. Wald test was used to calculate *p*-values and *p*-values were adjusted for multiple testing with the Benjamini-Hochberg procedure [49, 50].

### TaqMan-PCR

Quantitative TaqMan real-time PCR was performed with the Rotor-Gene 6000 realtime rotary analyser (Quiagen, Hilden, Germany). MiRNA expression was assessed using the TaqMan^®^ MicroRNA Assays from Applied Biosystems following the manufacturer’s instructions. Total RNA was transcribed to cDNA using the TaqMan^®^ MicroRNA Reverse Transcription Kit (Applied Biosystems, Catalog # 4366597). cDNAs were amplified using the TaqMan^®^ Fast Universal PCR Master Mix (2X), no AmpErase^®^ UNG (Applied Biosystems, Catalog # 4352042). Single TaqMan-PCRs measurements were done for hsa-miR-21-5p (Assay ID 000397, Catalog # 4427975), hsa-miR-375 (Assay ID 000564, Catalog # 4427975), hsa-miR-215-5p (Assay ID 000518, Catalog # 4427975), hsa-miR-378a (Assay ID 002243, Catalog # 4427975) and U6 snRNA (Assay ID 001973, Catalog # 4427975) as a control. Thermal cycling conditions were initial 10 min at 95° C to activate the AmpliTaq^®^ Fast DNA Polymerase followed by 40 cycles of 95° C for 15 s and 60° C for 60 s. Results were normalized to the spliceosomal snRNA U6 and were analysed using ∆∆Ct method [51].

## SUPPLEMENTARY MATERIALS TABLES




