# Supplementary material for: Extensive screening of microRNA populations identifies hsa-miR-375 and hsa-miR-133a-3p as selective markers for human rectal and colon cancer
Source: Oncotarget. 2018 Jun 5;9(43):27256–67. doi: 10.18632/oncotarget.25535 (PMC6007480; doi:10.18632/oncotarget.25535)
Supplement: Supplementary file 1 [file oncotarget-09-27256-s001.pdf]

Extensive screening of microRNA populations identifies hsa-miR-375 and hsa-miR-133a-3p as selective markers for human rectal and colon cancer

SUPPLEMENTARY MATERIALS

Supplementary Table 1: Significantly expressed miRNAs in colon and rectal cancer tissues when compared to normal tissue at P_{adj} < 0.05 and a mean expression ≥ 10 read counts. The table illustrates a direct comparison for rectal cancer vs. colon cancer tissue of miRNAs significantly differentially expressed in rectal cancer (upper part), colon cancer (in the middle) or in both cancer types (lower part). Sections with significant deregulation were highlighted. Illustrations of these results are visualized in the matrix diagram (see also Figure 1). See Supplementary_Table_1

Supplementary Table 2: Clinical characteristics of patients with colon or rectal cancer

#	Entity	Sex	Age	Grading	T*	N*	M*	UICC
1	colon	m	46	2	3	0	0	IIA
2	colon	m	60	2	2	0	0	I
3	colon	f	70	2	3	1b	0	IIIB
4	colon	m	68	2	3	0	0	IIA
5	colon	m	54	3	1	0	0	I
6	colon	m	41	2	2	0	0	I
7	colon	m	76	3	3	1a	1b	IV
8	colon	f	71	2	3	0	0	IIA
9	colon	m	79	2	4b	1a	0	IIIC
10	colon	m	44	3	3	2a	0	IIIB
11	colon	m	73	2	2	1a	0	IIIA
12	colon	m	63	2	1	0	0	I
13	colon	f	64	3	3	1b	0	IIIB
14	colon	f	84	2	3	0	0	IIA
15	colon	f	67	2	3	1c	0	IIIB
16	rectum	f	44	2	3	0	0	IIA
17	rectum	m	80	2	2	0	0	I
18	rectum	f	50	2	3	2b	0	IIIC
19	rectum	f	55	2	2	0	0	I
20	rectum	m	61	2	4a	2a	0	IIIC
21	rectum	f	77	2	3	1a	0	IIIB
22	rectum	f	45	2	3	0	0	II
23	rectum	m	71	3	3	0	0	IIA
24	rectum	f	67	2	3	1c	0	IIIB
25	rectum	m	58	3	2	2a	0	IIIB
26	rectum	m	48	2	3	0	0	IIA
27	rectum	m	76	2	1	0	0	I
28	rectum	f	65	2	3	2a	1a	IVA
29	rectum	f	62	2	3	2a	0	IIIB

30	rectum	f	50	2	3	1b	1a	IVA
31	rectum	f	70	3	3	1	0	III
32	rectum	m	59	2	3	1a	1a	IV
33	rectum	m	49	2	2	0	0	I
34	rectum	f	76	2	3	1a	0	IIIB
35	rectum	f	67	2	4a	0	0	IIB
36	rectum	m	64	2	2	0	0	I
37	rectum	m	76	2	0	0	0	I
38	rectum	f	52	3	4b	2	1b	IV
39	rectum	f	77	2	2	0	0	I
40	rectum	f	55	3	4b	1a	1	IV
41	rectum	m	57	2	3	0	0	IIA
42	rectum	m	47	2	1	1a	0	IIIA
43	rectum	m	81	2	3	0	0	IIA
44	rectum	f	42	2	2	1	0	IIIA
45	rectum	m	46	2	3	1b	1a	IV
46	rectum	m	82	2	2	0	0	I
47	rectum	m	76	2	2	1	0	IIIA
48	rectum	m	52	2	2	1a	0	IIIA
49	rectum	f	61	2	3	0	1a	IVA
50	rectum	f	80	2	2	0	0	I

The table shows additional clinical data of patients including tumor entity, sex and age of the patients, tumor grading, TNM stage according to the TNM Classification of Malignant Tumors (Primary tumor (T), regional lymph nodes (N), distant metastasis (M)) and UICC stage.
