# Supplementary material for: Extensive screening of microRNA populations identifies hsa-miR-375 and hsa-miR-133a-3p as selective markers for human rectal and colon cancer
Source: Oncotarget. 2018 Jun 5;9(43):27256–67. doi: 10.18632/oncotarget.25535 (PMC6007480; doi:10.18632/oncotarget.25535)
Supplement: Supplementary file 2 [file oncotarget-09-27256-s002.docx]

|  |  | **Colon Cancer** | | |  |  | **Rectal Adenocarcinoma** | | |
| --- | --- | --- | --- | --- | --- | --- | --- | --- | --- |
| **name** | **MIMAT** | **baseMean** | **log2FoldChange** | **padj*** | **significant in** | **name** | **baseMean** | **log2FoldChange** | **padj*** |
| hsa-miR-375 | MIMAT0000728 | 4892 | -0.59 | 3.82E-01 | **Rectum** | **hsa-miR-375** | **2002** | **-1.76** | **1.35E-09** |
| hsa-miR-181c-3p | MIMAT0004559 | 39 | 0.87 | 1.42E-01 | **Rectum** | **hsa-miR-181c-3p** | **16** | **0.93** | **5.74E-05** |
| hsa-miR-181c-5p | MIMAT0000258 | 327 | 0.65 | 1.35E-01 | **Rectum** | **hsa-miR-181c-5p** | **125** | **0.73** | **2.50E-04** |
| hsa-miR-214-3p | MIMAT0000271 | 342 | 0.62 | 2.57E-01 | **Rectum** | **hsa-miR-214-3p** | **167** | **1.00** | **6.46E-04** |
| hsa-miR-421 | MIMAT0003339 | 54 | 0.69 | 1.69E-01 | **Rectum** | **hsa-miR-421** | **20** | **1.06** | **1.04E-03** |
| hsa-miR-29c-5p | MIMAT0004673 | 71 | -0.84 | 1.02E-01 | **Rectum** | **hsa-miR-29c-5p** | **24** | **-0.98** | **2.20E-03** |
| hsa-miR-92a-3p | MIMAT0000092 | 16681 | 0.63 | 8.13E-02 | **Rectum** | **hsa-miR-92a-3p** | **7554** | **0.77** | **2.33E-03** |
| hsa-miR-455-3p | MIMAT0004784 | 89 | 0.57 | 4.55E-01 | **Rectum** | **hsa-miR-455-3p** | **49** | **1.09** | **3.30E-03** |
| hsa-miR-138-5p | MIMAT0000430 | 26 | -0.43 | 6.02E-01 | **Rectum** | **hsa-miR-138-5p** | **13** | **-1.21** | **3.30E-03** |
| hsa-miR-30e-5p | MIMAT0000692 | 3587 | -0.39 | 3.78E-01 | **Rectum** | **hsa-miR-30e-5p** | **1537** | **-0.58** | **4.85E-03** |
| hsa-miR-483-3p | MIMAT0002173 | 12 | -1.04 | 2.85E-01 | **Rectum** | **hsa-miR-483-3p** | **15** | **1.69** | **5.33E-03** |
| hsa-miR-452-5p | MIMAT0001635 | 35 | 0.66 | 8.91E-02 | **Rectum** | **hsa-miR-452-5p** | **13** | **0.64** | **5.33E-03** |
| hsa-miR-27a-3p | MIMAT0000084 | 6518 | 0.60 | 1.87E-01 | **Rectum** | **hsa-miR-27a-3p** | **2279** | **0.72** | **5.35E-03** |
| hsa-miR-194-3p | MIMAT0004671 | 110 | -0.29 | 5.15E-01 | **Rectum** | **hsa-miR-194-3p** | **39** | **-0.93** | **7.98E-03** |
| hsa-miR-10b-5p | MIMAT0000254 | 49450 | -0.60 | 4.44E-01 | **Rectum** | **hsa-miR-10b-5p** | **11323** | **-0.85** | **9.56E-03** |
| hsa-miR-194-5p | MIMAT0000460 | 23438 | -0.37 | 4.50E-01 | **Rectum** | **hsa-miR-194-5p** | **5891** | **-0.82** | **1.40E-02** |
| hsa-miR-214-5p | MIMAT0004564 | 30 | 0.21 | 7.59E-01 | **Rectum** | **hsa-miR-214-5p** | **17** | **0.80** | **1.44E-02** |
| hsa-miR-450a-5p | MIMAT0001545 | 16 | 0.67 | 3.82E-01 | **Rectum** | **hsa-miR-450a-5p** | **16** | **0.89** | **1.49E-02** |
| hsa-miR-190a-5p | MIMAT0000458 | 136 | 0.10 | 9.28E-01 | **Rectum** | **hsa-miR-190a-5p** | **36** | **-0.83** | **1.72E-02** |
| hsa-miR-130b-3p | MIMAT0000691 | 76 | 0.35 | 3.72E-01 | **Rectum** | **hsa-miR-130b-3p** | **29** | **0.64** | **1.76E-02** |
| hsa-miR-92b-3p | MIMAT0003218 | 414 | 0.71 | 1.01E-01 | **Rectum** | **hsa-miR-92b-3p** | **144** | **0.69** | **1.90E-02** |
| hsa-miR-199b-5p | MIMAT0000263 | 352 | -0.11 | 8.52E-01 | **Rectum** | **hsa-miR-199b-5p** | **247** | **0.70** | **2.59E-02** |
| hsa-miR-365a/b-3p | MIMAT0000710 | 251 | 0.01 | 9.96E-01 | **Rectum** | **hsa-miR-365a/b-3p** | **94** | **0.89** | **2.65E-02** |
| hsa-miR-4532 | MIMAT0019071 | 458 | 0.27 | 8.26E-01 | **Rectum** | **hsa-miR-4532** | **1004** | **-1.38** | **2.79E-02** |
| hsa-miR-192-5p | MIMAT0000222 | 108400 | -0.47 | 3.69E-01 | **Rectum** | **hsa-miR-192-5p** | **34401** | **-0.75** | **3.25E-02** |
| hsa-miR-200a-5p | MIMAT0001620 | 192 | 0.01 | 9.96E-01 | **Rectum** | **hsa-miR-200a-5p** | **52** | **-0.81** | **3.33E-02** |
| hsa-miR-455-5p | MIMAT0003150 | 25 | -0.25 | 7.61E-01 | **Rectum** | **hsa-miR-455-5p** | **12** | **0.59** | **3.49E-02** |
| hsa-miR-18a-5p | MIMAT0000072 | 62 | 0.57 | 3.94E-01 | **Rectum** | **hsa-miR-18a-5p** | **21** | **0.80** | **3.53E-02** |
| hsa-let-7i-5p | MIMAT0000415 | 7637 | 0.64 | 1.18E-01 | **Rectum** | **hsa-let-7i-5p** | **4631** | **0.64** | **3.86E-02** |
| hsa-miR-152-3p | MIMAT0000438 | 432 | 0.16 | 7.94E-01 | **Rectum** | **hsa-miR-152-3p** | **242** | **0.46** | **3.98E-02** |
| hsa-miR-125b-5p | MIMAT0000423 | 2069 | 0.58 | 5.28E-01 | **Rectum** | **hsa-miR-125b-5p** | **1199** | **0.75** | **4.03E-02** |
| hsa-miR-660-5p | MIMAT0003338 | 93 | -0.15 | 7.89E-01 | **Rectum** | **hsa-miR-660-5p** | **41** | **-0.43** | **4.44E-02** |
| hsa-miR-30c-5p | MIMAT0000244 | 4785 | -0.33 | 5.58E-01 | **Rectum** | **hsa-miR-30c-5p** | **1976** | **-0.48** | **4.75E-02** |
| hsa-miR-199a-5p | MIMAT0000231 | 3048 | 0.26 | 7.79E-01 | **Rectum** | **hsa-miR-199a-5p** | **2756** | **0.82** | **4.76E-02** |
| hsa-miR-199a/b-3p | MIMAT0000232 | 36995 | 0.35 | 5.39E-01 | **Rectum** | **hsa-miR-199a/b-3p** | **20161** | **0.50** | **4.79E-02** |
| hsa-miR-148b-5p | MIMAT0004699 | 38 | 0.86 | 6.52E-02 | **Rectum** | **hsa-miR-148b-5p** | **14** | **0.46** | **4.79E-02** |
| hsa-miR-126-3p | MIMAT0000445 | 1415 | -0.43 | 2.87E-01 | **Rectum** | **hsa-miR-126-3p** | **497** | **-0.52** | **4.82E-02** |
| **hsa-miR-92a-1-5p** | **MIMAT0004507** | **40** | **1.13** | **9.12E-04** | **Colon** | hsa-miR-92a-1-5p | 12 | 0.75 | 5.72E-02 |
| **hsa-miR-493-5p** | **MIMAT0002813** | **53** | **1.40** | **9.12E-04** | **Colon** | hsa-miR-493-5p | 48 | 0.19 | 8.02E-01 |
| **hsa-miR-95-3p** | **MIMAT0000094** | **84** | **1.24** | **1.84E-03** | **Colon** | hsa-miR-95-3p | 21 | 0.26 | 5.18E-01 |
| **hsa-miR-181a-3p** | **MIMAT0000270** | **48** | **0.93** | **4.05E-03** | **Colon** | hsa-miR-181a-3p | 22 | 0.49 | 1.25E-01 |
| **hsa-miR-3656** | **MIMAT0018076** | **296** | **-1.83** | **9.96E-03** | **Colon** | hsa-miR-3656 | 155 | 0.46 | 5.89E-01 |
| **hsa-miR-381-3p** | **MIMAT0000736** | **53** | **-0.85** | **1.10E-02** | **Colon** | hsa-miR-381-3p | 23 | -0.11 | 8.60E-01 |
| **hsa-miR-17-3p** | **MIMAT0000071** | **27** | **0.97** | **1.15E-02** | **Colon** | hsa-miR-17-3p | 12 | 0.59 | 1.41E-01 |
| **hsa-miR-136-3p** | **MIMAT0004606** | **46** | **-0.74** | **1.32E-02** | **Colon** | hsa-miR-136-3p | 27 | -0.24 | 6.03E-01 |
| **hsa-miR-133a-3p** | **MIMAT0000427** | **620** | **-1.25** | **1.58E-02** | **Colon** | hsa-miR-133a-3p | 393 | -0.10 | 9.05E-01 |
| **hsa-miR-301a-3p** | **MIMAT0000688** | **47** | **1.18** | **1.76E-02** | **Colon** | hsa-miR-301a-3p | 14 | 0.47 | 1.06E-01 |
| **hsa-miR-7-5p** | **MIMAT0000252** | **5625** | **1.25** | **1.84E-02** | **Colon** | hsa-miR-7-5p | 1632 | 0.16 | 7.99E-01 |
| **hsa-miR-203a-3p** | **MIMAT0000264** | **1551** | **1.11** | **2.70E-02** | **Colon** | hsa-miR-203a-3p | 530 | 0.43 | 3.72E-01 |
| **hsa-miR-378g** | **MIMAT0018937** | **26** | **-1.31** | **3.10E-02** | **Colon** | hsa-miR-378g | 11 | -0.78 | 1.75E-01 |
| **hsa-miR-1247-3p** | **MIMAT0022721** | **47** | **1.47** | **3.10E-02** | **Colon** | hsa-miR-1247-3p | 12 | 0.52 | 2.95E-01 |
| **hsa-miR-550a-3p** | **MIMAT0003257** | **60** | **0.96** | **4.19E-02** | **Colon** | hsa-miR-550a-3p | 27 | 0.65 | 8.71E-02 |
| **hsa-miR-378a-5p** | **MIMAT0000731** | **151** | **-1.96** | **1.67E-09** | **Both** | **hsa-miR-378a-5p** | **56** | **-1.49** | **2.51E-08** |
| **hsa-miR-135b-5p** | **MIMAT0000758** | **65** | **3.02** | **1.79E-08** | **Both** | **hsa-miR-135b-5p** | **16** | **2.10** | **5.90E-08** |
| **hsa-miR-584-5p** | **MIMAT0003249** | **25** | **1.78** | **1.72E-06** | **Both** | **hsa-miR-584-5p** | **7** | **1.64** | **5.89E-08** |
| **hsa-miR-182-5p** | **MIMAT0000259** | **3273** | **1.98** | **1.62E-05** | **Both** | **hsa-miR-182-5p** | **853** | **1.22** | **1.80E-06** |
| **hsa-miR-31-5p** | **MIMAT0000089** | **436** | **2.63** | **1.62E-05** | **Both** | **hsa-miR-31-5p** | **86** | **1.34** | **1.04E-03** |
| **hsa-miR-215-5p** | **MIMAT0000272** | **3354** | **-1.45** | **3.47E-05** | **Both** | **hsa-miR-215-5p** | **1602** | **-1.39** | **9.06E-05** |
| **hsa-miR-129-5p** | **MIMAT0000242** | **20** | **-2.42** | **3.70E-05** | **Both** | **hsa-miR-129-5p** | **12** | **-1.22** | **9.56E-03** |
| **hsa-miR-21-5p** | **MIMAT0000076** | **53682** | **1.24** | **1.86E-04** | **Both** | **hsa-miR-21-5p** | **25361** | **0.99** | **1.80E-06** |
| **hsa-miR-139-5p** | **MIMAT0000250** | **77** | **-1.60** | **2.92E-04** | **Both** | **hsa-miR-139-5p** | **29** | **-1.19** | **3.40E-05** |
| **hsa-miR-195-5p** | **MIMAT0000461** | **1462** | **-1.12** | **6.78E-04** | **Both** | **hsa-miR-195-5p** | **487** | **-0.62** | **4.79E-02** |
| **hsa-miR-708-5p** | **MIMAT0004926** | **66** | **1.28** | **8.21E-04** | **Both** | **hsa-miR-708-5p** | **34** | **1.33** | **6.86E-07** |
| **hsa-miR-24-2-5p** | **MIMAT0004497** | **63** | **0.93** | **9.12E-04** | **Both** | **hsa-miR-24-2-5p** | **27** | **0.79** | **4.32E-05** |
| **hsa-miR-509-3p** | **MIMAT0002881** | **16** | **2.41** | **9.12E-04** | **Both** | **hsa-miR-509-3p** | **2** | **1.37** | **3.49E-02** |
| **hsa-miR-503-5p** | **MIMAT0002874** | **14** | **1.49** | **1.24E-03** | **Both** | **hsa-miR-503-5p** | **5** | **1.73** | **1.02E-08** |
| **hsa-miR-497-5p** | **MIMAT0002820** | **225** | **-1.10** | **1.24E-03** | **Both** | **hsa-miR-497-5p** | **96** | **-0.74** | **3.59E-03** |
| **hsa-miR-96-5p** | **MIMAT0000095** | **244** | **1.66** | **1.24E-03** | **Both** | **hsa-miR-96-5p** | **71** | **1.58** | **2.63E-04** |
| **hsa-miR-27a-5p** | **MIMAT0004501** | **85** | **1.22** | **1.46E-03** | **Both** | **hsa-miR-27a-5p** | **39** | **0.75** | **2.21E-02** |
| **hsa-miR-1247-5p** | **MIMAT0005899** | **100** | **1.77** | **1.57E-03** | **Both** | **hsa-miR-1247-5p** | **36** | **1.06** | **1.72E-02** |
| **hsa-miR-21-3p** | **MIMAT0004494** | **641** | **1.24** | **1.84E-03** | **Both** | **hsa-miR-21-3p** | **272** | **0.72** | **2.07E-02** |
| **hsa-miR-149-5p** | **MIMAT0000450** | **36** | **-1.07** | **1.84E-03** | **Both** | **hsa-miR-149-5p** | **19** | **-0.64** | **3.56E-02** |
| **hsa-miR-215-3p** | **MIMAT0026476** | **22** | **-1.59** | **3.08E-03** | **Both** | **hsa-miR-215-3p** | **7** | **-1.72** | **1.30E-04** |
| **hsa-miR-378a-3p** | **MIMAT0000732** | **20097** | **-1.39** | **4.14E-03** | **Both** | **hsa-miR-378a-3p** | **6932** | **-1.64** | **1.35E-09** |
| **hsa-miR-552-5p** | **MIMAT0026615** | **25** | **1.86** | **4.24E-03** | **Both** | **hsa-miR-552-5p** | **9** | **1.16** | **3.59E-03** |
| **hsa-miR-181d-5p** | **MIMAT0002821** | **230** | **1.60** | **4.36E-03** | **Both** | **hsa-miR-181d-5p** | **65** | **1.12** | **5.79E-07** |
| **hsa-miR-93-5p** | **MIMAT0000093** | **1329** | **0.80** | **4.98E-03** | **Both** | **hsa-miR-93-5p** | **447** | **0.61** | **6.16E-04** |
| **hsa-miR-224-5p** | **MIMAT0000281** | **120** | **1.54** | **5.48E-03** | **Both** | **hsa-miR-224-5p** | **54** | **1.96** | **3.85E-07** |
| **hsa-miR-147b** | **MIMAT0004928** | **43** | **-1.43** | **5.52E-03** | **Both** | **hsa-miR-147b** | **12** | **-1.52** | **3.90E-06** |
| **hsa-miR-504-5p** | **MIMAT0002875** | **13** | **-1.59** | **7.63E-03** | **Both** | **hsa-miR-504-5p** | **6** | **-1.28** | **2.86E-04** |
| **hsa-miR-378c** | **MIMAT0016847** | **114** | **-1.36** | **8.78E-03** | **Both** | **hsa-miR-378c** | **47** | **-1.47** | **1.80E-06** |
| **hsa-miR-183-5p** | **MIMAT0000261** | **1170** | **1.60** | **1.15E-02** | **Both** | **hsa-miR-183-5p** | **251** | **1.44** | **4.32E-05** |
| **hsa-miR-552-3p** | **MIMAT0003215** | **41** | **1.48** | **1.15E-02** | **Both** | **hsa-miR-552-3p** | **15** | **1.11** | **3.87E-03** |
| **hsa-miR-592** | **MIMAT0003260** | **35** | **1.51** | **1.15E-02** | **Both** | **hsa-miR-592** | **9** | **0.95** | **4.16E-02** |
| **hsa-miR-424-3p** | **MIMAT0004749** | **16** | **1.18** | **1.45E-02** | **Both** | **hsa-miR-424-3p** | **7** | **1.59** | **1.80E-06** |
| **hsa-miR-424-5p** | **MIMAT0001341** | **92** | **1.21** | **2.70E-02** | **Both** | **hsa-miR-424-5p** | **43** | **1.53** | **3.49E-06** |
| **hsa-miR-25-3p** | **MIMAT0000081** | **2008** | **0.66** | **3.26E-02** | **Both** | **hsa-miR-25-3p** | **823** | **0.43** | **3.84E-02** |
| **hsa-miR-20a-5p** | **MIMAT0000075** | **734** | **0.93** | **3.71E-02** | **Both** | **hsa-miR-20a-5p** | **231** | **0.84** | **1.72E-02** |
| **hsa-miR-29c-3p** | **MIMAT0000681** | **982** | **-0.84** | **3.79E-02** | **Both** | **hsa-miR-29c-3p** | **321** | **-0.70** | **2.42E-02** |
| **hsa-miR-223-3p** | **MIMAT0000280** | **1166** | **1.26** | **4.63E-02** | **Both** | **hsa-miR-223-3p** | **360** | **1.00** | **3.30E-03** |

* Wald test was used to calculate p-values and p-values were adjusted for multiple testing with the Benjamini-Hochberg procedure.

**Supplementary Table 1 (online available):** Significantly expressed miRNAs in colon and rectal cancer tissues when compared to normal tissue at *P*_adj_ < 0.05 and a mean expression ≥ 10 read counts. The table illustrates a direct comparison for rectal cancer vs. colon cancer tissue of miRNAs significantly differentially expressed in rectal cancer (upper part), colon cancer (in the middle) or in both cancer types (lower part). Sections with significant deregulation were highlighted. Illustrations of these results are visualized in the matrix diagram (see also Figure 1).
